# Stem cell therapy for intrauterine adhesions: a systematic review and meta-analysis of clinical outcomes

**DOI:** 10.1186/s12884-025-08271-y

**Published:** 2025-12-13

**Authors:** Feifei Yuan, Yun Zhang, Yanping Qian, Xiaoyu Zhou, Meiling Wang, Hongjuan Hu, Xiaojun Zhong, Hongping Niu

**Affiliations:** 1 Department of Gynecology, The First Clinical Medical College, Yunnan University of Chinese Medicine, 1076 Yuhua Road, Chenggong District, Kunming City, Yunnan Province 650500 China; 2 The First Clinical Medical College, Yunnan University of Chinese Medicine, 1076 Yuhua Road, Chenggong District, Kunming City, Yunnan Province 650500 China; 3 Department of Gynecology, The First Affiliated Hospital of Yunnan University of Chinese Medicine, 120 Guanghua Street, Wuhua District, Kunming City, Yunnan Province 650021 China; 4 Department of Medical Record Statistics, The First Affiliated Hospital of Yunnan University of Chinese Medicine, 120 Guanghua Street, Wuhua District, Kunming City, Yunnan Province 650021 China

**Keywords:** Stem Cell Therapy, Intrauterine Adhesions, Asherman's Syndrome, Endometrial Repair, Infertility, Live Birth, Clinical Pregnancy, Meta-analysis

## Abstract

**Objective:**

Intrauterine Adhesions (IUA), including Asherman's Syndrome, are a significant cause of female infertility, while stem cell interventions emerge as a promising therapeutic strategy. This meta-analysis aims to systematically evaluate the effectiveness of stem cell interventions for IUA by analyzing various pregnancy outcomes.

**Methods:**

This meta-analysis systematically searched five databases (PubMed, Web of Science, EMBASE, Scopus, and ProQuest) to identify clinical studies on stem cell interventions for IUA and endometrial repair. Data on cumulative live birth, biochemical pregnancy, clinical pregnancy, implantation rate, early spontaneous abortions, and ectopic pregnancy were extracted and analyzed. Subgroup analyses were conducted based on disease type and stem cell intervention method.

**Results:**

Out of 211 identified records, 10 studies were included. The overall pooled proportion of cumulative live birth was 0.40 (95% confidence interval (CI): 0.22 to 0.60), with significant heterogeneity (I^2^ = 46.2%). Subgroup analysis showed the highest live birth rates in "General IUA" (0.57) and with "Autologous Cells with Scaffold/Matrix" (0.5632). Controlled trials demonstrated a significant benefit of stem cell therapy for live birth (odds ratio (OR) = 2.2535, 95% CI: 1.1750 to 4.3221). The pooled proportion for cumulative biochemical pregnancy was 0.6053 (95% CI: 0.4445 to 0.7461) with no significant heterogeneity. For cumulative clinical pregnancy, the pooled proportion was 0.4605 (95% CI: 0.3425 to 0.5831), also with significant heterogeneity (I^2^ = 36.9%). "General Intrauterine Adhesion" (0.6250) and "Autologous Cells with Scaffold/Matrix" (0.6207) showed the highest clinical pregnancy rates. Stem cell interventions significantly improved clinical pregnancy rates compared to controls (OR = 3.1277, 95% CI: 1.3802 to 7.0877). The overall implantation rate was 0.1200 (95% CI: 0.0549 to 0.2424). Early spontaneous abortions occurred at a pooled proportion of 0.1705 (95% CI: 0.1055 to 0.2637), and ectopic pregnancies at 0.0568 (95% CI: 0.0238 to 0.1293), with no significant heterogeneity in either.

**Conclusion:**

Stem cell therapy appears to be a promising intervention for IUA and endometrial repair, particularly with autologous cells combined with a scaffold, though more high-quality, controlled trials are needed to confirm these findings and optimize treatment protocols.

## Introduction

IUA characterized by the formation of scar tissue within the uterine cavity, represent a significant cause of female infertility, oligomenorrhea, amenorrhea, and recurrent miscarriage [1–3]. These adhesions typically arise from endometrial damage, frequently induced by surgical procedures such as dilatation and curettage, but also stemming from pregnancy-related complications, infections, and other uterine surgeries [2]. While hysteroscopic surgery remains the gold standard for IUA treatment, often supplemented with post-operative strategies like intrauterine balloons or hormonal therapy, these conventional approaches frequently fall short in preventing recurrence and restoring fertility [1, 2, 4]. The inherent challenges in endometrial tissue repair due to the uterus's anatomical position further underscore the limitations of current treatments, prompting the search for more effective therapeutic modalities [2, 4].

In recent years, mesenchymal stem cells (MSCs) have emerged as a promising therapeutic avenue for IUA due to their remarkable self-renewal, immunomodulatory, anti-inflammatory, pro-angiogenic, and anti-fibrotic properties, which are crucial for tissue regeneration [1, 3, 5, 6]. MSCs have demonstrated potential in reversing conditions like Asherman's Syndrome and thin endometrium, thereby improving pregnancy outcomes and addressing infertility [7]. Furthermore, advancements in cell-free therapies, such as MSC-derived extracellular vesicles, are gaining traction as they offer the therapeutic benefits of MSCs without the limitations associated with direct cell transplantation, including concerns related to cell survival, engraftment, long-term efficacy, and manufacturing complexities [4, 5, 8–10].

Previous meta-analyses have begun to explore the role of stem cell therapy in IUA. For instance, Chen et al. [11] conducted a systematic review comparing autologous and allogeneic stem cell treatments in recurrent IUA patients. Their analysis of 10 studies (116 patients) primarily focused on improvements in endometrial thickness and pregnancy rates. They reported that autologous stem cell therapy led to a more significant increase in endometrial thickness (mean difference, 1.68; 95% CI: 1.30–2.07; *P* < 0.00001) and an improved pregnancy rate (relative risk, 1.55; 95% CI: 1.19–2.02, *P* < 0.001), with no serious adverse reactions observed. Similarly, other reviews have explored MSC applications and extracellular vesicles [4, 8–10]. While these previous studies provide valuable insights into the efficacy and safety of stem cell therapy, they have often been limited in their scope, focusing on a narrow set of outcomes and frequently lacking comprehensive, stratified subgroup analyses. Specifically, prior meta-analyses have not adequately addressed the heterogeneity in outcomes by rigorously considering factors such as the severity and specific type of uterine pathology, nor have they thoroughly investigated the impact of different stem cell intervention methods (cell source, scaffold use, direct infusion) on a broader spectrum of critical pregnancy outcomes.

To address these gaps, the present meta-analysis aims to provide a more comprehensive evaluation of stem cell interventions for IUA. We intend to specifically focus on key fertility and pregnancy outcomes including cumulative live birth, cumulative biochemical pregnancy, cumulative clinical pregnancy, implantation rate, early spontaneous abortion, and ectopic pregnancy. Furthermore, a detailed subgroup analysis will be conducted, stratifying outcomes based on the disease type and severity as well as the specific stem cell intervention method employed, to better understand the sources of heterogeneity and provide more nuanced clinical guidance.

## Methods

This meta-analysis was conducted in accordance with the Preferred Reporting Items for Systematic Reviews and Meta-Analyses (PRISMA) guidelines [12] and the recommendations outlined in the Cochrane Handbook for Systematic Reviews of Interventions.

### Search strategy

A comprehensive systematic search was performed across five electronic databases: PubMed, Web of Science, EMBASE, Scopus, and ProQuest. The search strategy was developed using a combination of Medical Subject Headings (MeSH) terms and keywords related to stem cell therapy and uterine pathologies. The following search terms and their various combinations were utilized:

- Stem Cell Therapy: "Stem Cells" [MeSH], "Mesenchymal Stem Cells" [MeSH], "Hematopoietic Stem Cells" [MeSH], "Pluripotent Stem Cells" [MeSH], "Embryonic Stem Cells" [MeSH], "Induced Pluripotent Stem Cells" [MeSH], "Adult Stem Cells" [MeSH], "Menstrual Blood Stem Cells," "Bone Marrow Stem Cells," "Adipose Derived Stem Cells," "Umbilical Cord Stem Cells," "Cell Therapy," "Cell Transplantation," "Regenerative Medicine."
- Intrauterine Adhesions & Asherman's Syndrome: "Intrauterine Adhesions" [MeSH], "Asherman Syndrome" [MeSH], "Uterine Synechiae," "Endometrial Fibrosis," "Thin Endometrium," "Endometrial Atrophy."

Boolean operators ("AND," "OR") were used to combine these terms effectively. For instance, a general search string included combinations such as ("Stem Cells" OR "Mesenchymal Stem Cells" OR "Cell Therapy") AND ("Intrauterine Adhesions" OR "Asherman Syndrome"). The search was not restricted by publication date or language during the initial screening phase. The initial search identified a total of 165 records, with an additional 46 records found through other sources (e.g., manual searching of reference lists). After removing 32 duplicate records, 179 unique records proceeded to the screening phase.

### Selection criteria

Studies were included in the meta-analysis if they met the following criteria:

1. Population: Patients diagnosed with IUA or AS, including severe/refractory cases, recurrent IUA, or thin endometrium causing infertility.
2. Intervention: Any type of stem cell therapy, including but not limited to autologous (menstrual blood-derived, bone marrow mononuclear cells, adipose-derived stromal vascular fraction, bone marrow-derived mesenchymal stem cells, endometrial stem cells) or allogeneic (umbilical cord mesenchymal stem cells) cells, administered with or without a scaffold/matrix, and often supplemented with hormonal support.
3. Comparison: Studies with either a control group (standard care, placebo) or a pre-post intervention design (single-arm studies).
4. Outcomes: Studies reporting on at least one of the following fertility and pregnancy-related outcomes: cumulative live birth rate, cumulative biochemical pregnancy rate, cumulative clinical pregnancy rate, implantation rate, early spontaneous abortion rate, and ectopic pregnancy rate. Endometrial regeneration parameters (thickness, hysteroscopic appearance), menstrual volume, and safety were also considered.
5. Study Design: Randomized controlled trials (RCTs) were prioritized, followed by prospective cohort studies and other prospective interventional designs. Case reports and review articles were excluded. Clinical studies registered as clinical trials were given preference.

### Data extraction

Data extraction was performed independently by two reviewers. Any discrepancies were resolved through discussion until consensus was reached, or by arbitration by a third reviewer. The following data points were extracted from each included study:

- Study Characteristics: PMID, country of origin, study period, study design (prospective study, prospective cohort study, non-controlled prospective phase I clinical trial, unblinded pilot study, prospective single-arm longitudinal study, prospective single-arm study, single-center one-group pretest–posttest clinical trial, open-label randomized clinical trial, double-blinded randomized controlled trial, retrospective single-center interventional study).
- Patient Population: Specific disease type and severity (severe IUAs, Grade III-V) refractory to traditional treatments; severe Asherman's Syndrome; recurrent IUA causing secondary infertility or embryo transfer failure; severe Asherman's Syndrome with oligomenorrhea/amenorrhea and thin endometrium (< 5 mm); Asherman's Syndrome (Grade I-IV) or Endometrial Atrophy (ET < 5 mm); refractory Asherman's Syndrome (Grade III-V) with endometrial thickness < 6 mm; moderate to severe IUA (AFS score 5–12); recurrent IUA with infertility; recurrent moderate-to-severe IUA (AFS score ≥ 6); recurrent thin endometrium with moderate-to-severe IUA (AFS stage III or ESGE Grade III +)).
- Intervention Details:
  - ⚬ Stem Cell Source: Autologous (menstrual blood-derived stem cells, bone marrow mononuclear cells, adipose-derived stromal vascular fraction, bone marrow-derived mesenchymal stem cells, endometrial stem cells or Allogeneic.
  - ⚬ Delivery Method: Direct intrauterine infusion/implantation, or with a scaffold/matrix (collagen scaffold, sodium hyaluronate gel, collagen scaffold complex).
  - ⚬ Concomitant Treatments: Details of hormonal support (e.g., oral estradiol valerate, progesterone, Progynova, medroxyprogesterone acetate, dydrogesterone, acetylsalicylic acid).

- Endpoints: Primary and secondary endpoints as defined by each study (endometrial regeneration, ongoing pregnancy rate, IUA score improvement, live birth rate, clinical pregnancy rate, biochemical pregnancy rate, miscarriage rate, menstrual volume, IVF feasibility, safety).
- Inclusion and Exclusion Criteria: Detailed criteria for patient selection in each study.
- Quantitative Data for Meta-analysis: For each outcome, the number of observations (patients/pregnancies) and the number of events (e.g., cumulative live births, clinical pregnancies) were extracted.

### Bias assessment

The risk of bias for each included study was independently assessed by two reviewers using the domains specified in the Cochrane Handbook for Systematic Reviews of Interventions. The following domains were evaluated: random sequence generation, allocation concealment, blinding of participants and personnel, blinding of outcome assessment, incomplete outcome data, selective reporting, and other sources of bias. Each domain was rated as "low risk," "high risk," or "unclear risk" of bias. Discrepancies in bias assessment were resolved through discussion to reach a consensus. The overall risk of bias for each study was determined based on these individual domain assessments.

### GRADE evidence assessment

The quality of evidence for key outcomes was assessed using the Grading of Recommendations Assessment, Development and Evaluation (GRADE) methodology. Two independent reviewers rated the evidence for each outcome based on five domains: risk of bias, inconsistency, indirectness, imprecision, and publication bias. Discrepancies were resolved by a third reviewer.

### Statistical analyses

Statistical analyses were performed using R statistical software, following best practices for meta-analysis [13]. For cumulative live birth, biochemical pregnancy, clinical pregnancy, implantation rate, early spontaneous abortions, and ectopic pregnancy, pooled proportions were calculated using both fixed-effect and random-effects models. For controlled trials, pooled ORs for cumulative live birth and cumulative clinical pregnancy were calculated.

Heterogeneity among studies was assessed using Cochran's Q test and quantified by the I^2^ statistic. An I^2^ value of 0% to 40% was considered to indicate low heterogeneity, 30% to 60% moderate heterogeneity, 50% to 90% substantial heterogeneity, and 75% to 100% considerable heterogeneity. A *p*-value less than 0.10 for the Q test or an I^2^ value greater than 50% suggested significant heterogeneity, prompting the use of a random effects model; otherwise, a fixed-effect model was employed. This approach was applied to all primary outcomes.

Subgroup analyses were conducted to investigate potential sources of heterogeneity for cumulative live birth and cumulative clinical pregnancy. Subgroups were defined by disease type and specific stem cell intervention method, as detailed in the Data Extraction section. Differences between subgroups were assessed using a chi-squared test (Q-test). All results are presented with 95% confidence intervals (CIs). A two-sided *p*-value less than 0.05 was considered statistically significant. Forest plots were generated to visually represent the pooled estimates and the results of subgroup analyses.

## Results

**Fig. 1:**
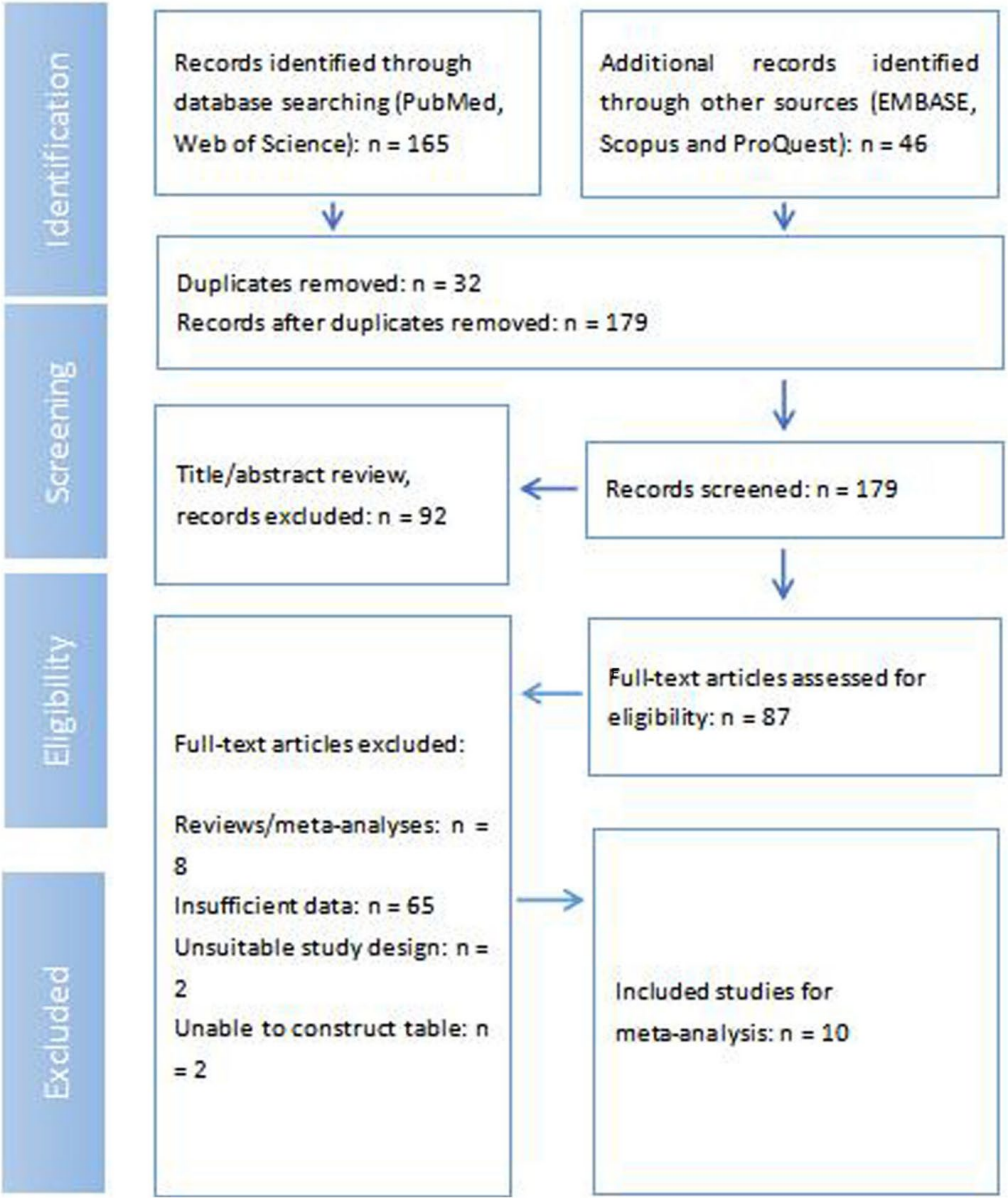
Flow diagram of study selection process

The meta-analysis was conducted following a systematic search of PubMed, Web of Science, EMBASE, Scopus, and ProQuest databases, identifying a total of 165 records and 46 additional records. After removing 32 duplicates, 179 records proceeded to the screening phase. Ninety-two records were excluded during title and abstract review, leaving 87 full-text articles for eligibility assessment. Of these, 8 were reviews/meta-analyses, 65 had insufficient data, 2 had unsuitable study designs, and 2 were unable to construct a table from, leading to their exclusion. Ultimately, 10 studies were included in the meta-analysis (Fig. 1).

### Characteristics of included studies

The meta-analysis incorporated data from ten clinical studies on stem cell interventions for IUA and endometrial repair, all conducted in China except for one in South Korea [14] and one in India [15]. The studies primarily consisted of prospective designs, including prospective studies [16], prospective cohort studies [17], non-controlled prospective phase I clinical trials [18], unblinded pilot studies [14], prospective single-arm longitudinal studies [15], prospective single-arm studies [19], and single-center, one-group pretest–posttest clinical trials [20]. Notably, two studies were randomized controlled trials (RCTs): an open-label RCT [21] and a double-blinded RCT [22], offering higher levels of evidence. One study was a retrospective, single-center interventional study [23]. The target patient populations suffered from various forms of IUA and AS, ranging from severe/refractory cases to recurrent IUA causing infertility, moderate-to-severe IUA, and thin endometrium. Interventions predominantly involved autologous stem cells, including menstrual blood-derived stem cells [16, 19], bone marrow mononuclear cells [17], adipose-derived stromal vascular fraction [14], bone marrow-derived mesenchymal stem cells [21, 23], and endometrial stem cells [20]. Two studies utilized allogeneic stem cells: umbilical cord mesenchymal stem cells [18] and human umbilical cord mesenchymal stem cells [22]. Many experimental groups received hormonal support alongside stem cell therapy. Key primary endpoints varied but commonly included endometrial regeneration (measured by thickness, hysteroscopy, or biopsy), ongoing pregnancy rate, or IUA score improvement, while secondary endpoints often assessed other pregnancy outcomes (live birth, clinical pregnancy, biochemical pregnancy), menstrual volume, and safety. Inclusion criteria generally targeted infertile women with severe, refractory, or recurrent IUA/AS, with specific age ranges and exclusion of confounding factors like active infections or genetic abnormalities (Table 1).

**Table 1:** Characteristics of included studies

Study	Country	Study Period	Study Design	Disease	Intervention drugs	Primary endpoint	Secondary endpoint	Inclusion Criteria	Exclusion Criteria
Tan et al., 2016 [16]	China	Ethical approval year: 2012	Prospective study	Severe IUAs (Grade III-V) refractory to traditional treatments	Experimental group: Autologous menstrual blood-derived stem cells (menSCs) + oral estradiol valerate (4–6 mg/day) and progesterone (40 mg IM if needed)	Endometrial regeneration (measured by endometrial thickness via ultrasound)	Pregnancy outcomes (natural or assisted) post-therapy	Women aged 20–40 with severe IUAs (Grade III-V) refractory to standard treatments	Active genital tuberculosis (TB)
Zhao et al., 2016 [17]	China	Ethical approval year: 2012	Prospective cohort study	Severe Asherman's Syndrome	Experimental group: Autologous bone marrow mononuclear cells (BMNCs) + Progynova (6 mg/day) and progesterone (60 mg injection) ​Control group: No intervention (natural controls)	Endometrial regeneration (assessed by hysteroscopy and biopsy)	Restoration of menstrual function and endometrial thickness	Women with severe AS diagnosed by hysteroscopy, normal hormone profiles and karyotypes	N/A
Cao et al., 2014 [18]	China	Follow-up until August 2017	non-controlled prospective phase I clinical trial	Recurrent IUA causing secondary infertility or embryo transfer failure	Experimental group: Umbilical cord mesenchymal stem cells (UC-MSCs) + Progynova (estradiol 6 mg/day) and progesterone (60 mg injection)	IUA score and maximum endometrial thickness	Live birth rate, ongoing pregnancy rate (> 12 weeks), miscarriage rate, menstrual volume, and endometrial histological changes	Women < 45 years with recurrent IUA-related infertility/transfer failure, desiring pregnancy	Hysteroscopy contraindications, karyotype abnormalities, uterine malformations, severe adenomyosis, pregnancy contraindications, estradiol contraindications, pelvic malignancy/radiotherapy history
Lee et al., 2019 [14]	South Korea	July 2015 to May 2017	Unblinded pilot study	Severe Asherman's Syndrome with oligomenorrhea/amenorrhea and thin endometrium (< 5 mm)	Experimental group: Autologous adipose-derived stromal vascular fraction (AD-SVF) + hormone therapy (oral estradiol valerate 6 mg/day + medroxyprogesterone acetate 10 mg/day)	Change in endometrial thickness (EMT) post-treatment	Clinical pregnancy rate	Women aged 20–44 with severe AS, failed prior hysteroscopic adhesiolysis + hormone therapy, BMI < 30 kg/m^2^, and AMH ≥ 1.0 ng/mL	N/A
Singh et al., 2019 [15]	India	January 2013 to January 2014 (with 5-year follow-up)	Prospective single-arm longitudinal study, registered as CTRI/2013/08/003896	Asherman's Syndrome (Grade I-IV) or Endometrial Atrophy (ET < 5 mm)	Experimental group: Autologous bone marrow-derived mononuclear stem cells (MNCs) + oral estradiol valerate (2 mg thrice daily) and medroxyprogesterone (10 mg/day)	Endometrial regeneration (menstrual resumption/improvement and increased endometrial thickness)	IVF feasibility (if ET > 7 mm) and long-term endometrial status (5-year follow-up)	Infertile women aged 24–38 with AS/EA refractory to standard treatments (hysteroscopic adhesiolysis + estrogen therapy)	Active genital TB, chronic/hematopoietic diseases affecting bone marrow, or unwillingness to participate
Ma et al., 2020 [19]	China	Ethical approval year: 2017	Prospective single-arm study	Refractory Asherman's Syndrome (Grade III-V) with endometrial thickness < 6 mm	Experimental group: Autologous menstrual blood-derived stem cells (MenSCs) + hormone replacement therapy (HRT: oral estradiol 2 mg thrice daily ± dydrogesterone 10 mg/day)	Endometrial regeneration (improvement in endometrial thickness and morphology via ultrasound)	Pregnancy outcomes (biochemical/clinical pregnancy rates post-embryo transfer)	Women aged 22–40 with refractory AS (Grade III-V), < 6 mm endometrial thickness after standard treatment, and > 1 year of infertility	Congenital/inherited infertility causes, acute urogenital infections, or other genital diseases
Zhu et al., 2023 [21]	China	February 2016 to January 2020	Open-label randomized clinical trial, registered as NCT02680366	Moderate to severe IUA (AFS score 5–12)	Experimental group: Autologous bone marrow-derived mesenchymal stem cells (BMSCs) seeded on collagen scaffold + Foley balloon catheter. ​Control group: Foley balloon catheter alone	Ongoing pregnancy rate (fetal heartbeat confirmed until ≥ 12 gestational weeks)	Menstrual volume (PBAC scores) and improvement. Endometrial thickness (MET) and increase. IUA recurrence rate (second-look hysteroscopy). AFS score reduction. Pregnancy outcomes (miscarriage, preterm birth, ectopic pregnancy, live birth). Pregnancy/neonatal complications (e.g., placenta previa, FGR)	Women aged 20–40 years with moderate/severe IUA (AFS 5–12), reproductive intention, normal ovarian function, BMI < 30 kg/m^2^	Thin endometrium without adhesions, congenital uterine malformations. Contraindications to bone marrow collection, pregnancy, or hormonal therapy. Prior hysteroscopic adhesiolysis > 3 times, chromosomal abnormalities. Adenomyosis/myoma (uterus > 50 gestational days size), malignancy history
Wang et al., 2024 [23]	China	January 2017 to January 2020	Retrospective, single-center interventional study	Recurrent IUA with infertility	Experimental group: Autologous bone marrow-derived mesenchymal stem cells (BM-MSCs, 2 × 10⁷ cells/mL) via intrauterine perfusion	Endometrial repair, menstrual flow improvement	Endometrial thickness (ultrasound). Menstrual flow (PBAC score). Reproductive outcomes. IUA recurrence (AFS score)	Infertile women with recurrent IUA (confirmed by hysteroscopy), ≥ 1 prior hysteroscopic adhesiolysis, failed estrogen/progesterone therapy, no contraindications to procedures/hormones	Active infections (hepatitis/HIV/syphilis), severe allergies. Contraindications to bone marrow aspiration or estrogen/progesterone. Recent blood transfusion (within 3 months)
Peng et al., 2025 [20]	China	2018 to 2020	Single-center, one-group pretest–posttest clinical trial, registered as CHICTR1800016769	Recurrent moderate-to-severe IUA (AFS score ≥ 6) with infertility	Experimental group: Autologous endometrial stem cells (1 × 10⁷ cells in 1 mL plasma) + 12.5% sodium hyaluronate gel, with hormonal support (Oestradiol valerate 3 mg/day + acetylsalicylic acid 50 mg/day)	Improvement in endometrial thickness/IUA severity	Menstrual recovery. IUA degree (AFS score) via hysteroscopy. Pregnancy outcomes (HCG confirmation, follow-up). RNA sequencing analysis of stem cells	Women aged 20–40 with recurrent moderate/severe IUA (AFS ≥ 6), infertility/multiple failed transplants, normal organ function, no infections, and reproductive intent	Chromosomal abnormalities, adenomyosis, malignancy. Severe physical/mental illness, surgical infections, coagulation dysfunction
Hou et al., 2025 [22]	China	August 2022 to November 2023	Single-center, double-blinded randomized controlled trial, registered as NCT05495711	Recurrent thin endometrium with moderate-to-severe IUA (AFS stage III or ESGE Grade III +)	Experimental group: hUC-MSC/collagen scaffold (CS) complex (2 × 10⁷ cells in 2 mL iECM) + standard hormone therapy (estradiol valerate + progesterone). ​Control group: Saline/CS placebo + standard hormone therapy	Cumulative live birth rate (cLBR, ≥ 28 weeks gestation)	Pregnancy outcomes (biochemical/clinical pregnancy, implantation, ongoing pregnancy, miscarriage, ectopic pregnancy rates). Endometrial parameters (thickness [EmT], pattern [EmP]). Psychological assessment (SCL-90 scores). Safety (laboratory tests, ECG, obstetrical/perinatal complications)	Women aged 20–39 with infertility, severe IUA (AFS ≥ III/ESGE ≥ III), failed prior ART (≥ 4 good-quality embryos stored), EmT < 6.5 mm despite high-dose estrogen, and consent	Untreated conditions affecting pregnancy (e.g., hydrosalpinx, endometriosis ≥ III, adenomyosis, large fibroids/tumors). Malignancies, infections (HIV/hepatitis), or contraindications to surgery/hormones. Pituitary tumors or uterine malformations

### Bias assessment of included studies

**Fig. 2:**
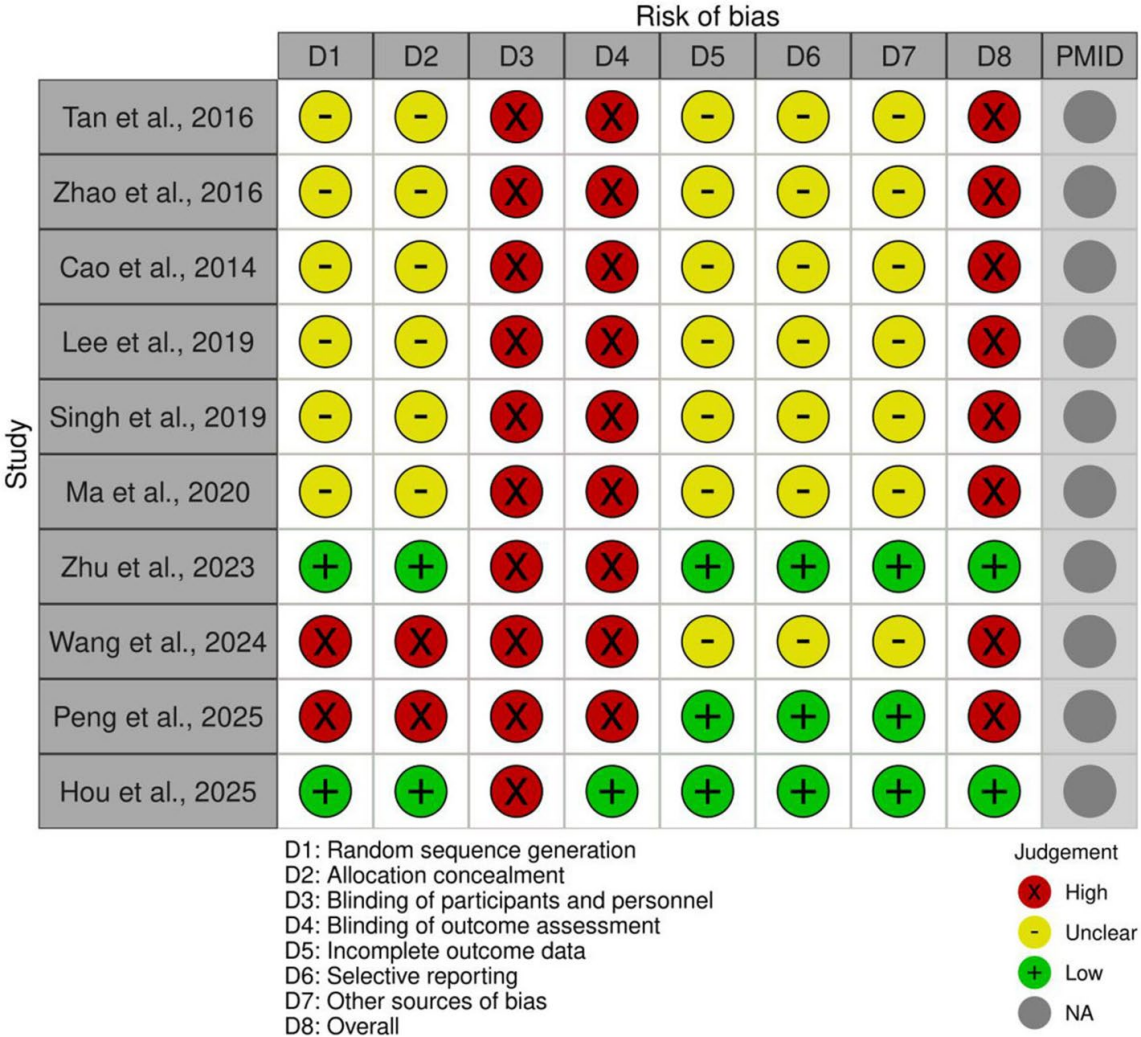
Bias assessment of included studies

The risk of bias assessment, shown as Fig. 2, revealed notable limitations across the ten included studies, impacting the overall methodological quality of the evidence. A significant majority of studies exhibited a high risk of bias in several critical domains. Specifically, the methods for random sequence generation and allocation concealment were largely unclear for most trials, raising concerns about potential selection bias. Similarly, information regarding incomplete outcome data and selective reporting was frequently unclear, indicating a potential for reporting biases. All studies were assessed as having a high risk of bias concerning the blinding of participants and personnel, suggesting that participants and researchers were aware of the assigned interventions. While the blinding of outcome assessment was also predominantly rated as high risk, indicating potential detection bias, there were some exceptions. Notably, only two studies, Zhu et al., 2023 and Hou et al., 2025, demonstrated a low risk of bias for random sequence generation, allocation concealment, incomplete outcome data, and selective reporting. However, Hou et al., 2025 was still at low risk for blinding of outcome assessment, while Zhu et al., 2023 maintained a high risk in this area. Peng et al., 2025, despite having low risk in some domains, was ultimately rated as high risk overall. This pervasive high risk of bias across multiple domains suggests that the findings should be interpreted with caution, highlighting the need for more rigorously designed and reported randomized controlled trials in this field.

### Cumulative live birth

**Fig. 3:**
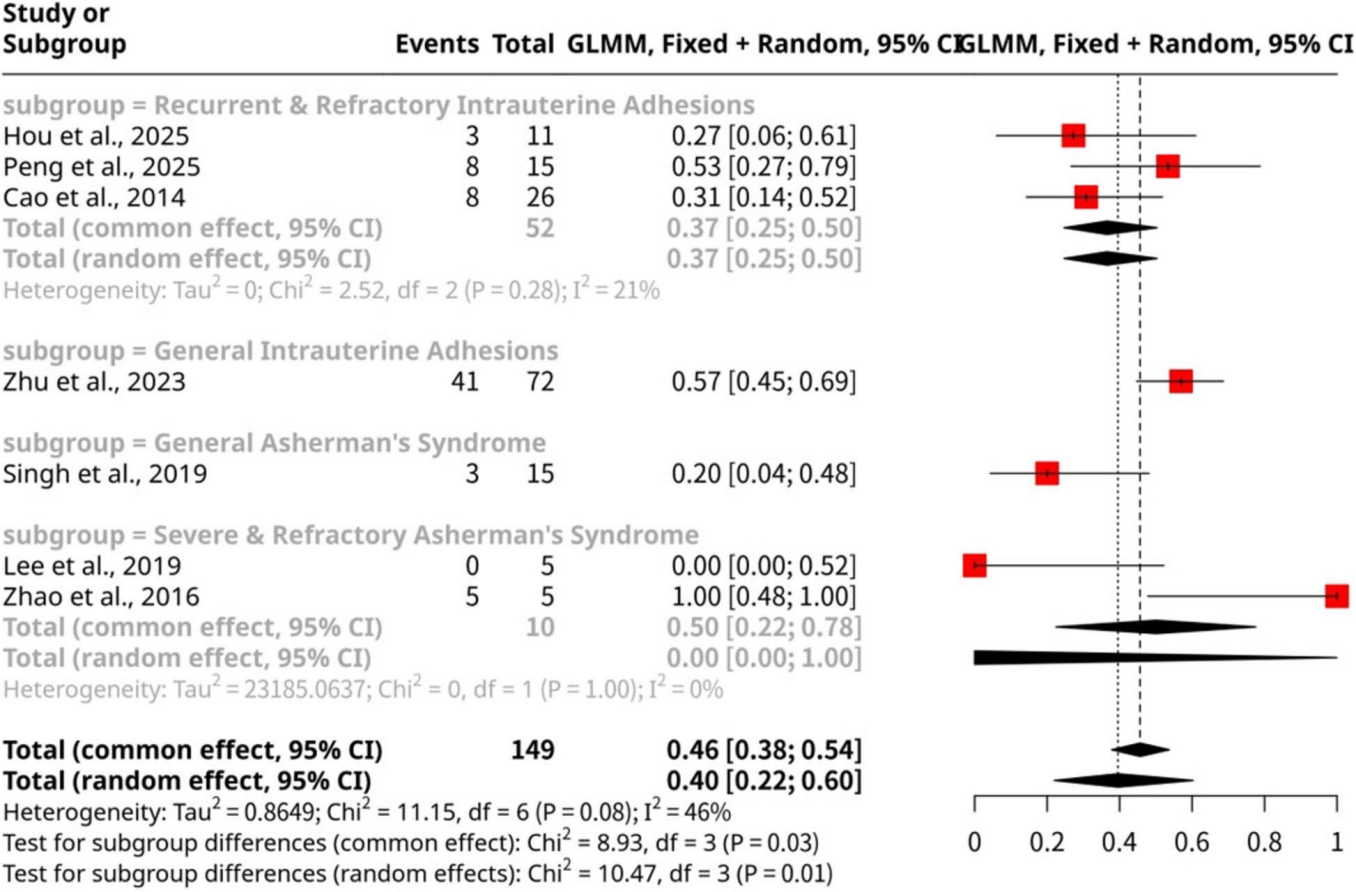
Forest plot of cumulative live birth proportions categorized by disease type

The meta-analysis of seven studies, involving 149 observations and 68 cumulative live birth events, investigated the effectiveness of stem cell therapy interventions (Fig. 3). The overall pooled proportion of cumulative live birth was 0.46 (95% CI: 0.38 to 0.54) using a common effect model, while a random effects model yielded a proportion of 0.40 (95% CI: 0.22 to 0.60). Significant heterogeneity was present across the included studies, with an I^2^ value of 46.2% (95% CI: 0.0% to 77.3%) and a statistically significant LRT test (*p*-value = 0.0002). Subgroup analysis, categorized by disease type under the common effect model, revealed notable variations in cumulative live birth rates: studies focusing on General IUA (k = 1) reported the highest proportion at 0.57 (95% CI: 0.45 to 0.68), followed by Severe & Refractory Asherman's Syndrome (k = 2) with 0.50 (95% CI: 0.22 to 0.78). In contrast, the Recurrent & Refractory IUA subgroup (k = 3) showed a proportion of 0.37 (95% CI: 0.25 to 0.50), and General Asherman's Syndrome (k = 1) had the lowest proportion at 0.20 (95% CI: 0.07 to 0.47). The statistically significant test for subgroup differences (common effect model: *p*-value = 0.0302; random effects model: *p*-value = 0.0149) suggests that the underlying disease category significantly influences the cumulative live birth rates following stem cell therapy.

**Fig. 4:**
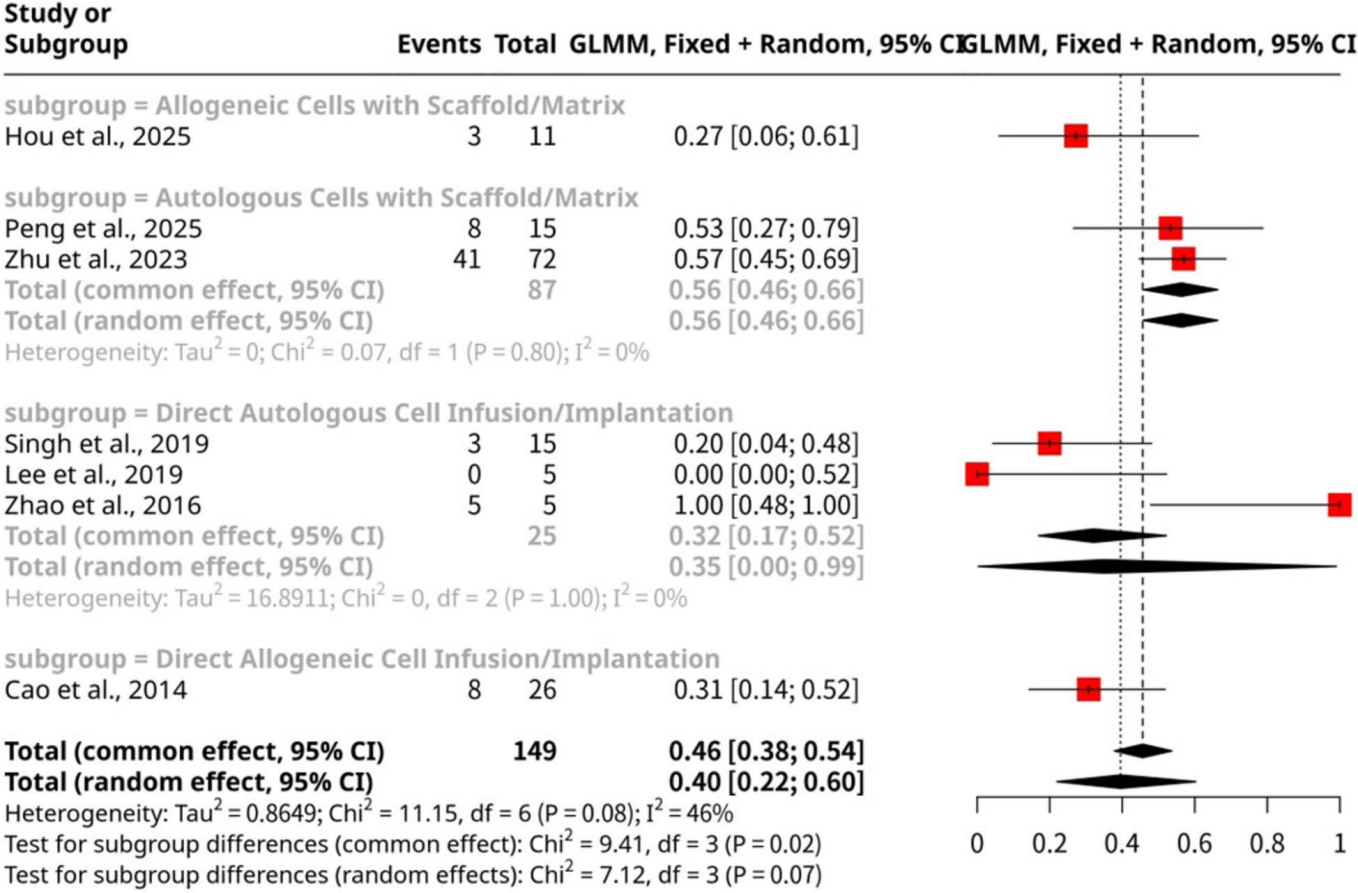
Forest plot of cumulative live birth proportions categorized by the specific stem cell intervention method

Subgroup analysis, shown as Fig. 4, categorized by the specific stem cell intervention method, revealed statistically significant differences in cumulative live birth outcomes (Q = 9.41, d.f. = 3, *p*-value = 0.0244 under the common effect model). The highest pooled proportion of cumulative live births was observed in the Autologous Cells with Scaffold/Matrix subgroup, which included two studies (Peng et al., 2025; Zhu et al., 2023), demonstrating a proportion of 0.5632 (95% CI: 0.4577 to 0.6633). This finding suggests that combining autologous cells with a supportive scaffold may optimize the regenerative environment for improved live birth rates. In contrast, Direct Autologous Cell Infusion/Implantation (Singh et al., 2019; Lee et al., 2019; Zhao et al., 2016) yielded a lower proportion of 0.3200 (95% CI: 0.1688 to 0.5216). Similarly, interventions involving allogeneic cells showed varying results: Direct Allogeneic Cell Infusion/Implantation (Cao et al., 2014) had a proportion of 0.3077 (95% CI: 0.1620 to 0.5055), while Allogeneic Cells with Scaffold/Matrix (Hou et al., 2025) resulted in a proportion of 0.2727 (95% CI: 0.0905 to 0.5857). These results indicate that the choice of stem cell source (autologous vs. allogeneic) and, more notably, the delivery method (direct infusion vs. scaffold-assisted) are crucial determinants of efficacy. The superior outcome with autologous cells combined with a scaffold implies a potential synergistic effect, enhancing cell retention and engraftment, thereby leading to better reproductive outcomes.

**Fig. 5:**
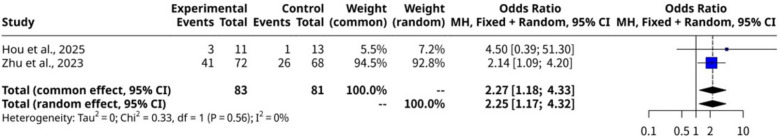
Forest plot of cumulative live birth odds ratios in controlled trials

The meta-analysis of two studies, shown as Fig. 5, encompassing 164 observations and 71 cumulative live birth events, investigated the effectiveness of stem cell interventions in comparison to control groups for cumulative live birth outcomes. The pooled ORs for cumulative live birth after stem cell intervention was 2.2658 (95% CI: 1.1843 to 4.3350) using a common effect model, indicating a statistically significant benefit of stem cell therapy over control groups (z = 2.47, *p*-value = 0.0135). The random effects model yielded a similar ORs of 2.2535 (95% CI: 1.1750 to 4.3221, z = 2.45, *p*-value = 0.0145). Importantly, there was no significant heterogeneity detected between the two included studies, with an I^2^ value of 0.0% and a non-significant Q-test for heterogeneity (Q = 0.33, d.f. = 1, *p*-value = 0.5632). This lack of heterogeneity suggests consistency in the observed treatment effect across the included trials, specifically Hou et al., 2025 and Zhu et al., 2023, both of which compared stem cell interventions to control groups for cumulative live birth.

### Cumulative biochemical pregnancy

**Fig. 6:**
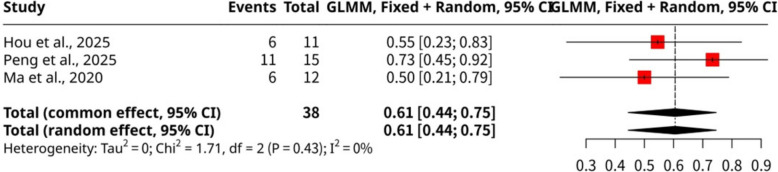
Forest plot of cumulative biochemical pregnancy

The meta-analysis of three studies, shown as Fig. 6, encompassing 38 observations and 23 cumulative biochemical pregnancy events, investigated the overall proportion of cumulative biochemical pregnancies following stem cell intervention. The pooled proportion of cumulative biochemical pregnancy was 0.6053 (95% CI: 0.4445 to 0.7461) using both common and random effects models. This indicates that over 60% of individuals achieved a biochemical pregnancy after stem cell therapy in the included studies. Importantly, the analysis revealed no significant heterogeneity among the studies, with an I^2^ value of 0.0% and non-significant *p*-values for both the Wald (*p*-value = 0.4260) and LRT (*p*-value = 0.4084) tests of heterogeneity. This consistency suggests a uniform effect across the studies by Hou et al., 2025, Peng et al., 2025, and Ma et al., 2020, regarding cumulative biochemical pregnancy outcomes after stem cell intervention.

### Cumulative clinical pregnancy

**Fig. 7:**
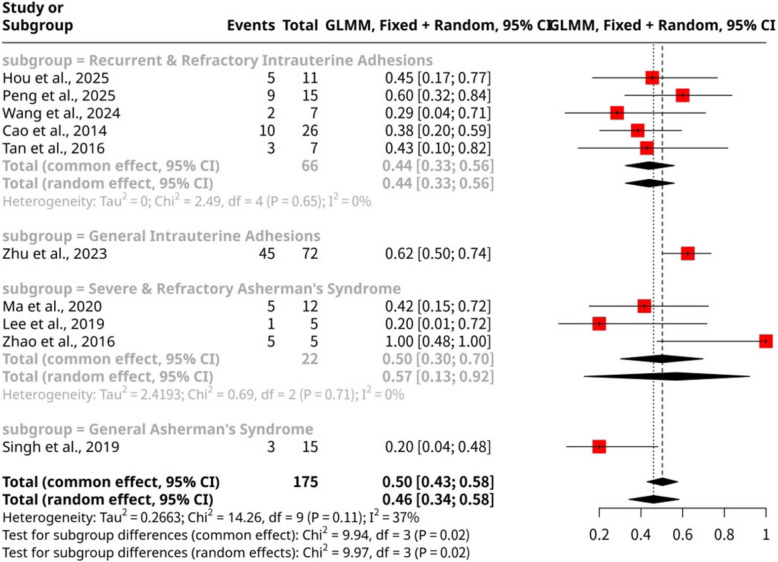
Forest plot of cumulative clinical pregnancy proportions categorized by disease type

The meta-analysis of ten studies, shown as Fig. 7, encompassing 175 observations and 88 cumulative clinical pregnancy events, assessed the overall proportion of cumulative clinical pregnancies following stem cell intervention. The pooled proportion was 0.5029 (95% CI: 0.4293 to 0.5763) under the common effect model, while the random effects model yielded 0.4605 (95% CI: 0.3425 to 0.5831). Significant heterogeneity was observed among the studies (I^2^ = 36.9%; LRT *p*-value = 0.0060), suggesting variability in outcomes that warrants further investigation. Subgroup analysis by disease type revealed notable differences (Test for subgroup differences: Q = 9.94, d.f. = 3, *p*-value = 0.0191). The highest proportion of cumulative clinical pregnancies was found in General IUA (Zhu et al., 2023), at 0.6250 (95% CI: 0.5084 to 0.7287). This was followed by Severe & Refractory Asherman's Syndrome (Ma et al., 2020; Lee et al., 2019; Zhao et al., 2016) with a proportion of 0.5000 (95% CI: 0.3024 to 0.6976), and Recurrent & Refractory IUA (Hou et al., 2025; Peng et al., 2025; Wang et al., 2024; Cao et al., 2014; Tan et al., 2016) at 0.4394 (95% CI: 0.3253 to 0.5603). Conversely, General Asherman's Syndrome (Singh et al., 2019) demonstrated the lowest proportion at 0.2000 (95% CI: 0.0659 to 0.4698). These findings highlight that the specific underlying uterine pathology significantly influences the success rate of stem cell interventions in achieving clinical pregnancy.

**Fig. 8:**
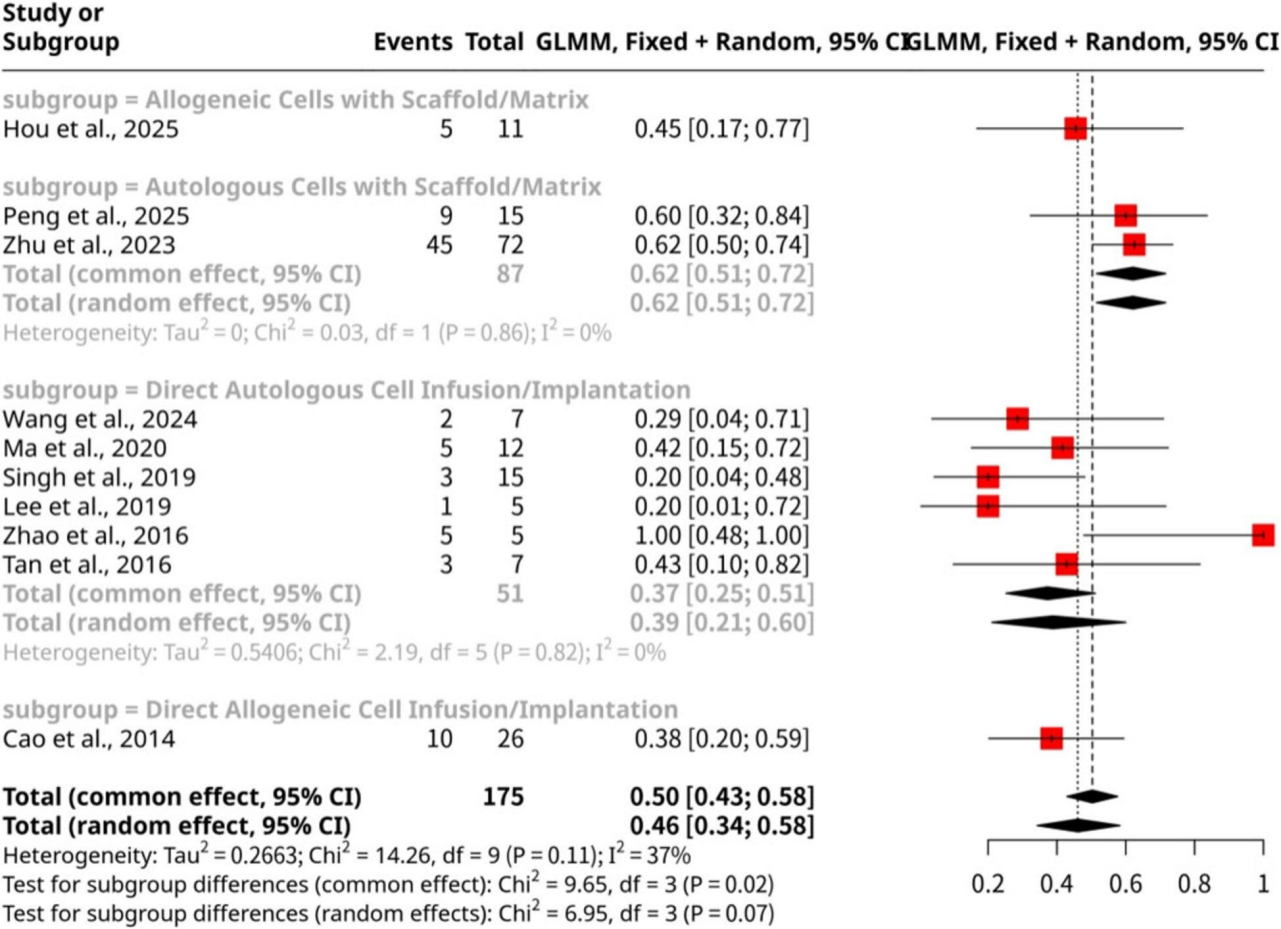
Forest Plot of cumulative clinical pregnancy proportions categorized by the specific stem cell intervention method

The meta-analysis of ten studies (*n* = 175 observations, 88 events), shown as Fig. 8, assessed the cumulative clinical pregnancy rate following stem cell interventions. Subgroup analysis, stratified by specific stem cell intervention methods, revealed statistically significant differences in cumulative clinical pregnancy rates (Test for subgroup differences: Q = 9.65, d.f. = 3, *p*-value = 0.0218). The highest proportion was observed in the Autologous Cells with Scaffold/Matrix subgroup (Peng et al., 2025; Zhu et al., 2023), achieving 0.6207 (95% CI: 0.5148 to 0.7162). This was notably higher than the Direct Autologous Cell Infusion/Implantation subgroup (Wang et al., 2024; Ma et al., 2020; Singh et al., 2019; Lee et al., 2019; Zhao et al., 2016; Tan et al., 2016), which yielded a proportion of 0.3725 (95% CI: 0.2518 to 0.5116). Interventions using allogeneic cells showed intermediate results: Allogeneic Cells with Scaffold/Matrix (Hou et al., 2025) had a proportion of 0.4545 (95% CI: 0.2028 to 0.7319), while Direct Allogeneic Cell Infusion/Implantation (Cao et al., 2014) resulted in 0.3846 (95% CI: 0.2210 to 0.5793). These findings suggest that the specific method of stem cell delivery, particularly the use of autologous cells with a scaffold, significantly impacts the success of achieving clinical pregnancy.

**Fig. 9:**
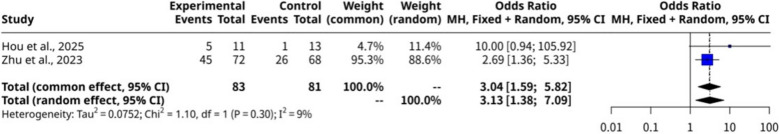
Forest plot of cumulative clinical pregnancy odds ratios in controlled trials

The meta-analysis of two controlled studies, shown as Fig. 9, encompassing 164 observations and 77 cumulative clinical pregnancy events, demonstrated the effectiveness of stem cell interventions compared to control groups. The pooled ORs for cumulative clinical pregnancy after stem cell intervention was 3.0393 (95% CI: 1.5884 to 5.8158) using a common effect model, indicating a statistically significant benefit of stem cell therapy (z = 3.36, *p*-value = 0.0008). The random effects model yielded a similar ORs of 3.1277 (95% CI: 1.3802 to 7.0877, z = 2.73, *p*-value = 0.0063). Importantly, the analysis showed very low heterogeneity between the two included studies, with an I^2^ value of 8.7% and a non-significant Q-test for heterogeneity (Q = 1.10, d.f. = 1, *p*-value = 0.2952). The studies, Hou et al., 2025 (Allogeneic Cells with Scaffold/Matrix for Recurrent & Refractory IUA) and Zhu et al., 2023 (Autologous Cells with Scaffold/Matrix for General IUA), both consistently reported a positive effect of stem cell therapy on cumulative clinical pregnancy outcomes.

### Implantation rate

**Fig. 10:**
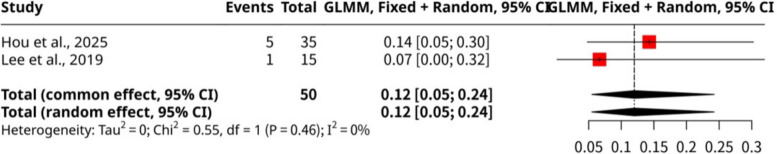
Forest plot of implantation rate proportions

The meta-analysis of two studies, shown as Fig. 10, involving 50 observations and 6 events, investigated the overall implantation rate following stem cell intervention. The pooled proportion for implantation was 0.1200 (95% CI: 0.0549 to 0.2424) under both common and random effects models. This indicates a relatively low but consistent implantation rate in the included studies. The analysis revealed no significant heterogeneity between the studies (I^2^ = 0.0%), with non-significant *p*-values for the Wald (*p*-value = 0.4582) and LRT (*p*-value = 0.4250) tests of heterogeneity. This consistency suggests a uniform effect across the included trials by Hou et al., 2025 and Lee et al., 2019, regarding the implantation rate after stem cell intervention.

### Early spontaneous abortions

**Fig. 11:**
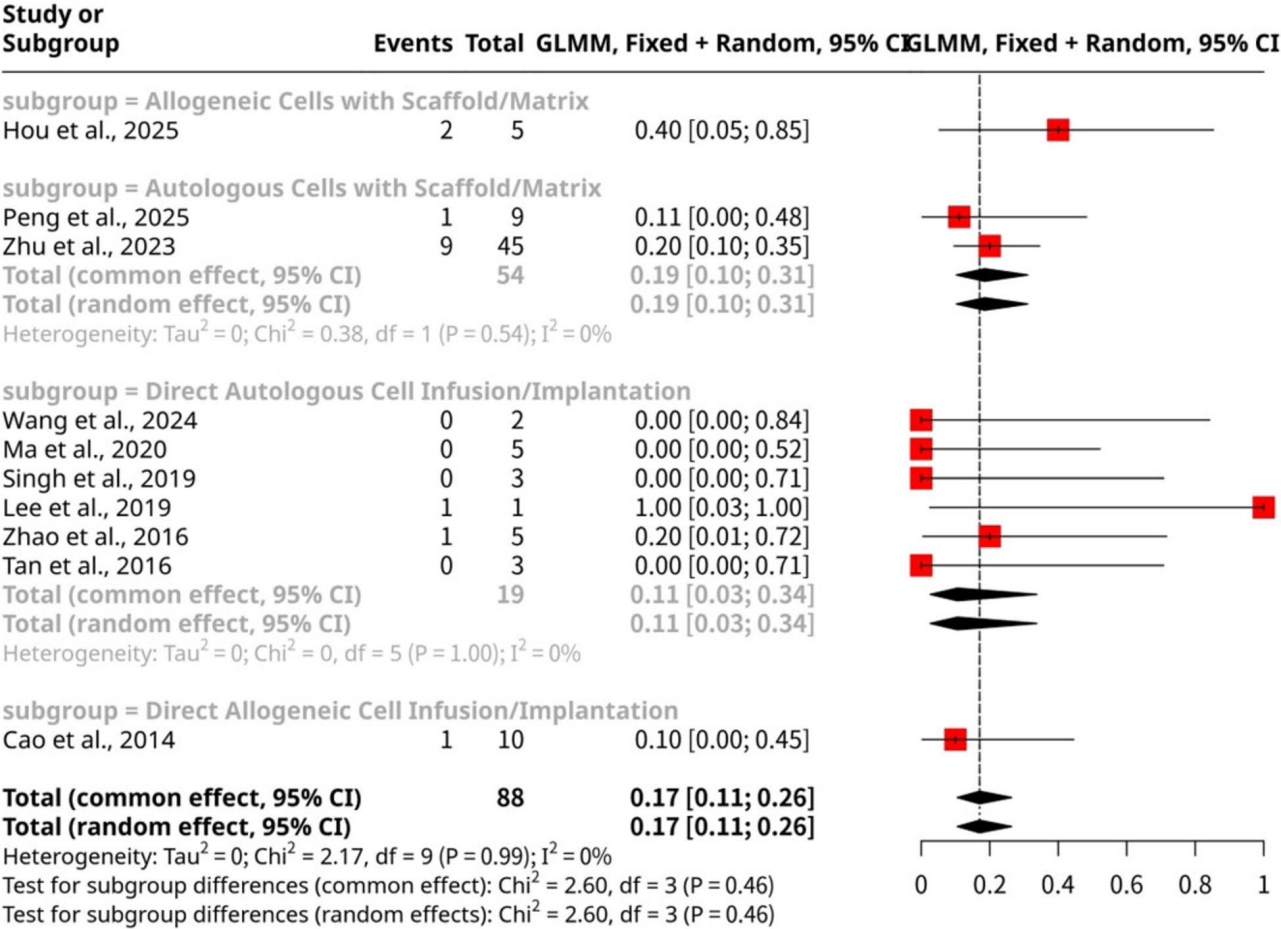
Forest plot of early spontaneous abortions proportions

The meta-analysis of ten studies, shown as Fig. 11, involving 88 observations and 15 early spontaneous abortion events, assessed the overall proportion of early spontaneous abortions following stem cell intervention. The pooled proportion was 0.1705 (95% CI: 0.1055 to 0.2637) under both common and random effects models, indicating an overall early spontaneous abortion rate of approximately 17%. No significant heterogeneity was detected across the studies (I^2^ = 0.0%; LRT *p*-value = 0.2888). Subgroup analysis, stratified by specific stem cell intervention methods, showed no statistically significant differences in early spontaneous abortion rates between the groups (Test for subgroup differences: Q = 2.60, d.f. = 3, *p*-value = 0.4570). Among the subgroups, Allogeneic Cells with Scaffold/Matrix (Hou et al., 2025) had the highest proportion of early spontaneous abortion at 0.4000 (95% CI: 0.1002 to 0.7996). This was followed by Autologous Cells with Scaffold/Matrix (Peng et al., 2025; Zhu et al., 2023) with a proportion of 0.1852 (95% CI: 0.1026 to 0.3111). Both Direct Autologous Cell Infusion/Implantation (Wang et al., 2024; Ma et al., 2020; Singh et al., 2019; Lee et al., 2019; Zhao et al., 2016; Tan et al., 2016) and Direct Allogeneic Cell Infusion/Implantation (Cao et al., 2014) showed lower proportions of 0.1053 (95% CI: 0.0265 to 0.3374) and 0.1000 (95% CI: 0.0139 to 0.4672), respectively.

### Ectopic pregnancy

**Fig. 12:**
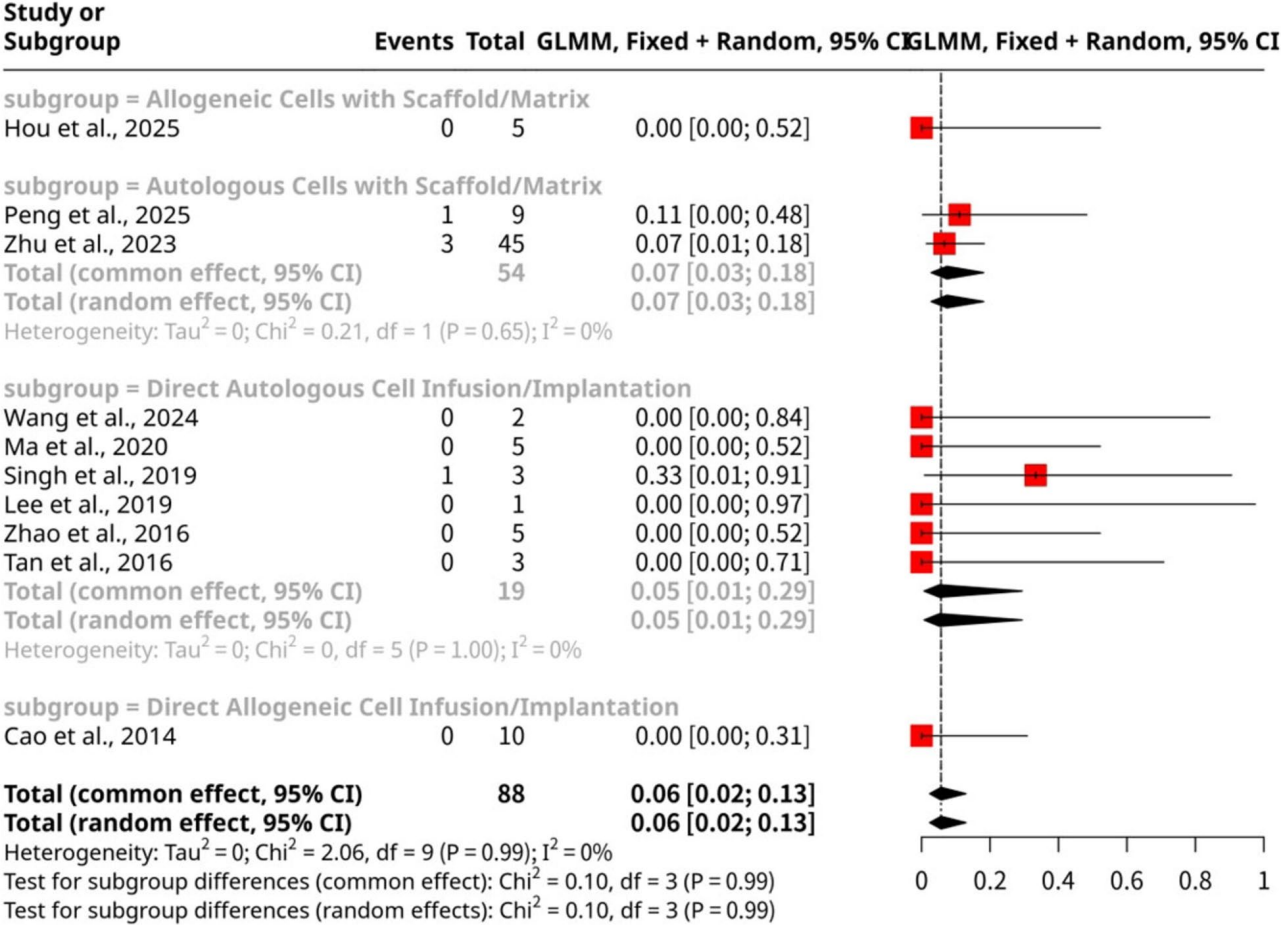
Forest plot of ectopic pregnancy proportions

The meta-analysis of ten studies, shown as Fig. 12, comprising 88 observations and 5 ectopic pregnancy events, investigated the overall proportion of ectopic pregnancies following stem cell intervention. The pooled proportion was 0.0568 (95% CI: 0.0238 to 0.1293) using both common and random effects models, indicating a relatively low incidence of ectopic pregnancies. The analysis revealed no significant heterogeneity among the studies (I^2^ = 0.0%), with non-significant *p*-values for the Wald (*p*-value = 0.9904) and LRT (*p*-value = 0.7149) tests of heterogeneity. Subgroup analysis, stratified by specific stem cell intervention methods, showed no statistically significant differences in ectopic pregnancy rates between the groups (Test for subgroup differences: Q = 0.10, d.f. = 3, *p*-value = 0.9918). The Autologous Cells with Scaffold/Matrix subgroup (Peng et al., 2025; Zhu et al., 2023) had a proportion of 0.0741 (95% CI: 0.0281 to 0.1813). The Direct Autologous Cell Infusion/Implantation subgroup (Wang et al., 2024; Ma et al., 2020; Singh et al., 2019; Lee et al., 2019; Zhao et al., 2016; Tan et al., 2016) showed a proportion of 0.0526 (95% CI: 0.0074 to 0.2939). Notably, both the Allogeneic Cells with Scaffold/Matrix (Hou et al., 2025) and Direct Allogeneic Cell Infusion/Implantation (Cao et al., 2014) subgroups reported proportions of 0.0000 (95% CI: 0.0000 to 1.0000), indicating no observed ectopic pregnancy events in these specific small subgroups. This overall low and consistent rate across intervention types suggests that stem cell therapies, regardless of specific method, do not appear to substantially increase the risk of ectopic pregnancy.

### GRADE evidence assessment

The overall quality of evidence for the effectiveness of stem cell therapy in treating Intrauterine Adhesions ranged from low to very low according to the GRADE approach, with the exception of the two controlled trials. Evidence for key outcomes like cumulative live birth rate and cumulative clinical pregnancy rate was assessed as low due to the high risk of bias in the majority of included studies, which were primarily single-arm or unblinded designs. The small sample sizes in many subgroups and the wide confidence intervals also contributed to imprecision. For outcomes with very few reported events, such as early spontaneous abortion, ectopic pregnancy, and implantation rate, the evidence quality was rated as very low, making it impossible to draw reliable conclusions. In contrast, the evidence from the two included controlled trials, which compared stem cell therapy to a control group, was of moderate quality. This was primarily because of consistent results (low heterogeneity) between these trials, despite their unblinded design and limited sample size, which led to a downgrade from high quality. These findings highlight the need for more rigorously designed, high-quality randomized controlled trials to provide definitive evidence on the efficacy and safety of stem cell interventions for IUA (Table 2).

**Table 2:** GRADE summary of findings for key outcomes

Outcome	No. of Datasets Included	Quality of Evidence	Justification
Cumulative Live Birth Rate	7 single-arm Datasets & 2 controlled Datasets	Low	Risk of Bias: The majority of studies were single-arm or unblinded, presenting a high risk of selection and detection bias. Although two RCTs were included, the overall evidence quality is downgraded by the high risk of bias in other studies.2. Imprecision: The overall confidence interval is wide (0.22 to 0.60), and subgroup analyses have small sample sizes, leading to imprecision.3. Publication Bias: N/A (Funnel plot or other analyses were not performed)
Cumulative Biochemical Pregnancy Rate	3 Datasets	Very Low	Risk of Bias: All included studies were single-arm or unblinded, indicating a high risk of bias.2. Imprecision: The number of included studies is very small (3), and the sample size is limited, making the results highly imprecise.3. Publication Bias: N/A (Sample size is too small to assess)
Cumulative Clinical Pregnancy Rate	10 Datasets	Low	Risk of Bias: The majority of studies were single-arm or unblinded, presenting a high risk of bias. The two controlled studies also showed some risk of bias.2. Imprecision: The overall confidence interval is wide (0.34 to 0.58), and subgroup analyses have small sample sizes.3. Publication Bias: N/A (Funnel plot or other analyses were not performed)
Cumulative Live Birth Rate (vs. Control)	2 controlled Datasets	Moderate	Downgrade: High Risk of Bias: Although these were RCTs, they were not blinded, introducing a risk of bias in outcome assessment.2. Upgrade: No Significant Heterogeneity: An I^2^ of 0.0% suggests high consistency across studies, which increases confidence in the findings.3. Imprecision: The number of studies is small (2), and the sample size is relatively limited.4. Publication Bias: N/A (Sample size is too small to assess)
Cumulative Clinical Pregnancy Rate (vs. Control)	2 controlled Datasets	Moderate	Downgrade: High Risk of Bias: Although these were RCTs, they were not blinded, introducing a risk of bias in outcome assessment.2. Upgrade: No Significant Heterogeneity: An I^2^ of 8.7% suggests high consistency across studies, which increases confidence in the findings.3. Imprecision: The number of studies is small (2), and the sample size is relatively limited.4. Publication Bias: N/A (Sample size is too small to assess)
Early Spontaneous Abortion Rate	10 Datasets	Very Low	Risk of Bias: The included studies had a high risk of bias in their design.2. Imprecision: The number of events (15) is very small, making it impossible to precisely estimate the risk.3. Publication Bias: N/A (Not assessed)
Ectopic Pregnancy Rate	10 Datasets	Very Low	Risk of Bias: The included studies had a high risk of bias in their design.2. Imprecision: The number of events (5) is extremely small, making it impossible to precisely estimate the risk.3. Publication Bias: N/A (Not assessed)
Implantation Rate	2 Datasets	Very Low	Risk of Bias: The included studies had a high risk of bias in their design.2. Imprecision: The number of studies is very small, and the number of events (6) is extremely limited, preventing reliable conclusions.3. Publication Bias: N/A (Sample size is too small to assess)

## Discussion

The meta-analysis reveals a significant difference in cumulative live birth outcomes from stem cell therapy across various disease categories, despite a moderate overall success rate. This difference is statistically significant (*p* < 0.05), underscoring that the specific disease and its severity are critical factors in treatment effectiveness. The General IUA subgroup (Zhu et al., 2023) showed the highest live birth proportion (0.57). This could be because these adhesions might be less severe or respond better to the combination of standard care with stem cell therapy, or due to the specific scaffold-based intervention used. In contrast, Recurrent & Refractory IUA (Hou et al., 2025; Peng et al., 2025; Cao et al., 2014) had a lower proportion (0.37). These cases are inherently tougher, often involving repeated treatment failures, high adhesion scores, and thin endometrium, which likely limit the full regenerative potential of stem cells. The General Asherman's Syndrome subgroup (Singh et al., 2019) had the lowest live birth rate (0.20), suggesting that broad AS diagnoses or less targeted treatments might yield poorer results. Conversely, Severe & Refractory Asherman's Syndrome (Lee et al., 2019; Zhao et al., 2016) showed a surprisingly higher proportion (0.50). This unexpected outcome in a more severe group might be due to highly targeted or potent stem cell types and delivery methods used in these studies, or specific patient selection criteria that favored better responders. However, the small sample sizes in some subgroups mean individual study characteristics can heavily influence these results. The overall heterogeneity (I^2^ = 46.2%) in the meta-analysis further points to varied results. Beyond the disease classifications, this variability likely stems from differences in the types of stem cells used (bone marrow-derived vs. adipose-derived), diverse intervention protocols (dosage, administration, co-treatments), and methodological differences among studies (study design, follow-up duration).

The meta-analysis also reveals a statistically significant disparity in cumulative live birth outcomes based on the specific stem cell intervention method employed, indicating that the delivery strategy and cell source are crucial determinants of efficacy. The Autologous Cells with Scaffold/Matrix subgroup (Peng et al., 2025; Zhu et al., 2023) exhibited the highest pooled live birth proportion of 0.5632. This superior outcome likely stems from several synergistic advantages. Scaffolds, such as collagen or hyaluronic acid gel, provide a crucial physical matrix that aids in the retention and localization of transplanted autologous cells at the site of uterine damage, preventing premature washout and ensuring higher cell engraftment. Furthermore, these scaffolds can mimic the natural extracellular environment, offering essential structural support and signaling cues that promote cell survival, proliferation, and differentiation. The autologous nature of the cells inherently minimizes the risk of immune rejection, allowing for better integration and long-term viability within the host tissue, which is vital for sustained regenerative processes leading to live birth. In contrast, Direct Autologous Cell Infusion/Implantation (Singh et al., 2019; Lee et al., 2019; Zhao et al., 2016) yielded a lower proportion of 0.3200. This reduced efficacy can primarily be attributed to the lack of a physical support system. Without a scaffold, directly infused or injected cells may be susceptible to rapid dispersion and washout from the uterine cavity due to natural physiological processes or fluid dynamics, thereby decreasing the number of cells that can successfully adhere and contribute to endometrial regeneration. The absence of a structured microenvironment might also hinder optimal cell-to-cell and cell-to-matrix interactions necessary for robust tissue repair.

The observed differences in outcomes across intervention methods are not surprising given the diverse nature of the stem cells themselves and their application protocols. The included studies employed a variety of cell sources, including autologous cells derived from menstrual blood [16, 19], bone marrow [21, 23], adipose tissue [14], and endometrium [20], as well as allogeneic cells from umbilical cord [18, 22]. Each cell type possesses a unique profile of growth factors, cytokines, and regenerative capabilities, which can influence their therapeutic potential. For instance, while bone marrow-derived cells are well-studied for their regenerative capacity, menstrual blood-derived stem cells are praised for their ease of collection and high proliferation rates. Furthermore, the delivery methods varied significantly, from direct intrauterine infusion [14, 15, 17, 19, 23] to using a scaffold [20–22]. These differences in cell source and delivery directly impact critical factors such as cell survival, retention, and engraftment within the hostile environment of a damaged uterus. The variability in these core therapeutic components makes it challenging to pinpoint a single "most effective" intervention, but our subgroup analysis points to the synergistic benefit of combining a high-quality cell source (autologous) with a method that optimizes cell delivery (scaffold). This highlights a critical need for future head-to-head trials to directly compare these different intervention strategies.

Beyond the variables we were able to analyze, such as disease type and intervention method, the studies included in this meta-analysis often did not adequately control for other potential confounding factors. These unmeasured variables could significantly impact the outcomes and complicate the interpretation of the results. For instance, patient-specific characteristics like age, the duration of infertility, and the number of previous failed treatments were not consistently reported or used as control variables. A woman's age and the long-standing nature of her fertility issues can independently influence live birth and clinical pregnancy rates, regardless of the treatment received. Similarly, the specific hormonal support protocols used alongside stem cell therapy, which varied widely among the studies, were difficult to standardize in the analysis. These uncontrolled variables may introduce additional layers of heterogeneity, making it challenging to isolate the true effect of the stem cell intervention itself. Future, more rigorous clinical trials must incorporate robust study designs that meticulously account for and control these confounding factors to provide a clearer picture of the therapy's true efficacy.

Interventions involving allogeneic cells, whether by Direct Allogeneic Cell Infusion/Implantation (Cao et al., 2014) or Allogeneic Cells with Scaffold/Matrix (Hou et al., 2025), generally showed lower proportions (0.3077 and 0.2727, respectively) compared to the autologous scaffold approach. A key factor contributing to this difference is the immunogenicity of allogeneic cells. Despite mesenchymal stem cells (MSCs) often being considered "immune-privileged," there remains a risk of host immune recognition and rejection, which can compromise cell survival, proliferation, and therapeutic function. While scaffolds might improve localized delivery for allogeneic cells, they might not fully overcome the challenges posed by immune responses. Additionally, variations in the specific type of allogeneic cell (umbilical cord vs. other sources), processing methods, and the inherent variability in donor cells could also contribute to the observed outcomes. Thus, the data suggest that optimizing both the stem cell source and the delivery platform is paramount. The synergistic effect of using autologous cells within a supportive scaffold appears to offer the most promising approach for improving cumulative live birth rates, likely by enhancing cell retention, viability, and the creation of a regenerative niche. This highlights the critical need for future research to further refine delivery techniques and explore the precise mechanisms underpinning these differential outcomes.

The meta-analysis of two controlled studies provides insights into the comparative effectiveness of stem cell interventions for cumulative live birth outcomes. Both included trials, Zhu et al., 2023 and Hou et al., 2025, demonstrated a statistically significant benefit of stem cell therapy over their respective control groups, contributing to a pooled OR of 2.2658 (95% CI: 1.1843 to 4.3350) and notably, showing no significant heterogeneity between them (I^2^ = 0.0%, *p*-value = 0.5632). Despite this consistency in relative efficacy, the two studies employed distinct stem cell intervention methods and addressed patient populations with varying disease characteristics. Zhu et al., 2023 utilized autologous bone marrow-derived mesenchymal stem cells (BMSCs) seeded on a collagen scaffold, along with a Foley balloon catheter, to treat patients with moderate to severe IUA. Their results indicated a significantly higher live birth rate in the stem cell group compared to the control receiving only a Foley catheter. In contrast, Hou et al., 2025 employed allogeneic human umbilical cord mesenchymal stem cells (hUC-MSCs) delivered via a collagen scaffold complex, focusing on a more refractory patient group suffering from recurrent thin endometrium and severe IUA, often with a history of failed assisted reproductive technologies. The striking similarity in the observed OR across these two studies, despite the differences in cell source (autologous vs. allogeneic) and the specific nature of the treated uterine pathology (general vs. refractory/recurrent adhesions), suggests that the scaffold-based delivery mechanism might be a critical common factor contributing to their comparable relative effectiveness. The use of a scaffold in both trials likely enhances cell retention, provides structural support for tissue regeneration, and creates a more conducive microenvironment for the transplanted cells, regardless of whether they are autologous or allogeneic. While autologous cells theoretically face fewer immune challenges, the scaffolding approach may sufficiently mitigate potential allogeneic immunogenicity or enhance engraftment to a degree where the relative benefit over control remains consistent across both scenarios. This consistency implies that the combined strategy of using cells with a physical scaffold might be a robust approach for improving cumulative live birth in different, though related, uterine conditions.

The meta-analysis of cumulative biochemical pregnancies, encompassing three studies (Hou et al., 2025; Peng et al., 2025; Ma et al., 2020), revealed remarkably low heterogeneity (I^2^ = 0.0%, *p*-values > 0.4 for heterogeneity tests). This striking consistency in the pooled proportion of 0.6053 for biochemical pregnancy across diverse interventions can be attributed to several converging factors from the included studies. Firstly, the definition of biochemical pregnancy (detection of hCG) is a clear and objective outcome measure, minimizing variability in reporting across studies. Secondly, all three studies specifically targeted patient populations suffering from severe and refractory uterine conditions, including recurrent thin endometrium with moderate-to-severe IUA (Hou et al., 2025), recurrent moderate-to-severe IUA (Peng et al., 2025), and refractory Asherman's Syndrome with thin endometrium (Ma et al., 2020). This shared underlying pathology of significant endometrial damage or dysfunction suggests that stem cell interventions may exert a consistent initial biological effect on the very early stages of pregnancy establishment within these challenging contexts. While the specific intervention methods varied (allogeneic cells with scaffold, autologous cells with scaffold, and direct autologous cell infusion), their common goal of enhancing endometrial receptivity and repair might lead to similar immediate impacts on biochemical indicators of pregnancy. Finally, with only three studies included, the statistical power to detect heterogeneity is inherently limited, meaning that even a low I^2^ value within a broad confidence interval [0.0%; 89.6%] should be interpreted with caution, as true underlying variability might exist but remains undetected.

Building upon the insights from live birth outcomes, the meta-analysis for cumulative clinical pregnancy similarly reveals a significant influence of both disease type and intervention method, albeit with some distinct patterns. The overall heterogeneity observed for clinical pregnancy (I^2^ = 36.9%; LRT *p*-value = 0.0060) is moderate, yet higher than that for live birth (I^2^ = 0.0%), suggesting that factors beyond the initial establishment of a pregnancy might contribute to variability at this later stage. When examining clinical pregnancy proportions by disease type, we see some parallels and divergences from live birth. The General IUA subgroup (Zhu et al., 2023) maintained the highest clinical pregnancy rate (0.6250), echoing its top performance in live birth. This consistency further supports the idea that less severe, more generalized IUA might be particularly responsive to stem cell therapy, potentially due to better underlying endometrial integrity or the specific autologous scaffold approach employed in this study. The Severe & Refractory Asherman's Syndrome subgroup (Ma et al., 2020; Lee et al., 2019; Zhao et al., 2016) showed a 0.5000 clinical pregnancy proportion, aligning with its relatively good live birth outcome, which, as previously discussed, might stem from highly targeted interventions or specific patient selection. However, the Recurrent & Refractory IUA subgroup (Hou et al., 2025; Peng et al., 2025; Wang et al., 2024; Cao et al., 2014; Tan et al., 2016) presented a lower clinical pregnancy proportion of 0.4394, consistent with the inherent challenges of these severe, repeatedly failed cases. The lowest clinical pregnancy rate was in General Asherman's Syndrome (Singh et al., 2019) at 0.2000, mirroring its lowest live birth rate, suggesting that broad diagnoses or less intensive approaches may yield limited success. The significant subgroup differences (*p*-value = 0.0191) underscore the need for tailored strategies based on specific uterine pathologies.

In terms of specific stem cell intervention methods, the pattern for clinical pregnancy closely tracks that of live birth outcomes. The Autologous Cells with Scaffold/Matrix subgroup (Peng et al., 2025; Zhu et al., 2023) again demonstrated the highest clinical pregnancy proportion of 0.6207. This further reinforces the hypothesis that combining autologous cells with a scaffold provides an optimal regenerative environment, leading to better early pregnancy establishment and progression. The sustained high performance across both clinical pregnancy and live birth suggests that this approach effectively supports not just initial implantation but also the subsequent development of a viable pregnancy. Conversely, Direct Autologous Cell Infusion/Implantation (Wang et al., 2024; Ma et al., 2020; Singh et al., 2019; Lee et al., 2019; Zhao et al., 2016; Tan et al., 2016) yielded a lower clinical pregnancy proportion of 0.3725, consistent with its lower live birth rate. This supports the argument that without a scaffold, transplanted cells face challenges with retention and stable engraftment, limiting their overall therapeutic impact on pregnancy outcomes. The allogeneic cell groups, both with and without scaffolds, also followed a similar trend to live birth, showing intermediate clinical pregnancy rates compared to the autologous scaffold group, likely due to factors such as immunogenicity or variations in cell viability and engraftment potential. The increased heterogeneity observed for clinical pregnancy compared to live birth (36.9% vs. 0.0% in controlled trials, 46.2% overall for live birth in the previous context not the controlled subgroup), warrants consideration. While biochemical pregnancy is a simple "yes/no" based on hCG, clinical pregnancy requires ultrasound confirmation, introducing more variables such as embryo quality, uterine receptivity, and potential early pregnancy losses. This suggests that while stem cells might consistently facilitate the *initial* biochemical detection, the journey to a confirmed clinical pregnancy might be more susceptible to other patient-specific factors, intervention nuances, and even differences in diagnostic criteria for clinical pregnancy across studies. The strong correlation between the efficacy patterns for clinical pregnancy and live birth highlights that successful early pregnancy establishment (biochemical and clinical) is a crucial prerequisite for live birth, and factors promoting the former are likely to influence the latter. Further research should focus on optimizing long-term cell survival and function to bridge any remaining gaps between clinical pregnancy and live birth outcomes.

The meta-analysis consistently revealed a lack of significant heterogeneity for both early spontaneous abortion (I^2^ = 0.0%, *p*-value = 0.2888) and ectopic pregnancy (I^2^ = 0.0%, *p*-value = 0.7149) across various stem cell intervention methods. This striking uniformity in outcomes, despite differences in cell source or delivery platforms, can be attributed to several factors. Firstly, both early spontaneous abortion and ectopic pregnancy are complex adverse events influenced by a multitude of factors, many of which lie beyond the direct or primary influence of uterine-targeted stem cell interventions. Early spontaneous abortions are frequently caused by embryonic chromosomal abnormalities or other intrinsic embryonic defects, which stem cell therapies cannot correct. While improved endometrial receptivity might reduce some environmentally-driven early losses, the inherent and often dominant genetic causes of early abortion are likely unaffected by variations in stem cell type or delivery, leading to a baseline rate that is difficult for intervention specifics to significantly alter. Similarly, ectopic pregnancies are primarily a consequence of impaired tubal transport or very specific, localized implantation errors outside the uterine cavity. Uterine-focused stem cell interventions, regardless of their method of delivery (direct infusion vs. scaffold), are unlikely to exert a substantial differential impact on these extra-uterine or highly localized initial implantation events. Secondly, the relatively low absolute number of events for both outcomes across the studies contributes to the observed lack of heterogeneity. With only 15 early spontaneous abortion events and 5 ectopic pregnancy events out of 88 observations, there is limited statistical power to detect subtle differences or variability across intervention types. The very low incidence of ectopic pregnancies, in particular, makes it challenging for any intervention or its variations to show a statistically discernible difference in its rate, especially when individual subgroups often consist of very few or zero events.

The observed lack of significant heterogeneity in early spontaneous abortion and ectopic pregnancy rates across different stem cell intervention methods, as discussed previously, warrants further exploration in light of the proposed mechanistic actions of stem cells. Preclinical and basic science studies extensively detail the multi-faceted mechanisms through which MSCs and their derivatives promote uterine repair. Studies highlight that MSCs exert their therapeutic effects primarily through anti-fibrotic [1, 24–26], immunomodulatory [1, 24], anti-inflammatory [24, 27], and pro-angiogenic properties [28]. Specific mechanisms involve inhibiting epithelial-mesenchymal transition [24, 25], promoting endometrial cell proliferation [28], and restoring the glandular and vascular architecture of the endometrium [24, 26]. Key signaling pathways modulated by stem cells or their exosomes include the TGF-β/Smad pathway (inhibited by specific miRNAs like miR-27b-3p, miR-145-5p, miR-16-5p from embryonic stem cell-derived Immunity-and-matrix-regulatory cells [24], the JAK2/STAT3 pathway (inhibited by kaempferol-UCMSC combination [25], the NF-κB pathway (regulated by miR-143 from placenta-derived MSC exosomes [26], and the VEGF/AKT/BAD pathway (activated by hydrogel-encapsulated adipose-derived stem cells and PRP [28]. These mechanistic insights consistently point towards the repair and regeneration of the uterine endometrium and the improvement of the intrauterine microenvironment.

When juxtaposed with clinical outcomes, this mechanistic focus can explain the observed homogeneity in certain adverse events. The primary targets of stem cell therapy are the local endometrial tissue and its receptivity, crucial for successful embryo implantation and early pregnancy maintenance [29]. However, ectopic pregnancy is largely a consequence of impaired tubal transport or very early, often extra-uterine, implantation errors. The uterine-focused mechanisms of stem cells, regardless of the specific type or delivery method (e.g., autologous vs. allogeneic, direct infusion vs. scaffold [11], are unlikely to significantly and differentially influence tubal function or the likelihood of an embryo implanting outside the uterus. Therefore, the lack of heterogeneity in ectopic pregnancy rates suggests that stem cell interventions in the uterus have a limited, if any, differential impact on this specific complication. Similarly, early spontaneous abortions are frequently attributed to embryonic chromosomal abnormalities, which are beyond the corrective capacity of endometrial-targeted stem cell therapies. While an improved uterine environment is undoubtedly beneficial, the fundamental genetic viability of the embryo remains a dominant factor. The consistent impact of various stem cell approaches on the uterine environment, coupled with the overriding influence of embryonic factors, likely contributes to a more uniform rate of early spontaneous abortions across different interventions. Furthermore, the overall low event rates for both ectopic pregnancy and early spontaneous abortion across the included studies may also limit the statistical power to detect any subtle differences or heterogeneity that might exist between various stem cell intervention modalities.

Based on our meta-analysis, stem cell therapy appears to be a promising intervention for IUA and endometrial repair. Our findings show that stem cell interventions significantly improve both live birth and clinical pregnancy rates compared to standard care, particularly when autologous cells are used with a scaffold or matrix. The pooled proportions for cumulative live birth and clinical pregnancy were 0.40 and 0.46, respectively, suggesting a substantial potential for restoring fertility in patients with refractory IUA. However, the quality of the current evidence, as assessed by the GRADE approach, is a critical limitation. The majority of included studies were single-arm or non-randomized, introducing a high risk of selection, performance, and detection bias. This widespread risk of bias, along with the imprecision stemming from small sample sizes and wide confidence intervals, downgraded the evidence for most outcomes to low or very low quality. While the two controlled trials provided moderate-quality evidence for the benefit of stem cell therapy, the overall lack of high-quality data necessitates caution. Future research must focus on conducting rigorously designed randomized controlled trials with larger patient cohorts, robust blinding protocols, and standardized outcome reporting to confirm our findings and establish definitive clinical guidelines.

Beyond the observed outcomes, a crucial aspect for optimizing stem cell therapy is the duration and frequency of administration. Our meta-analysis found a wide range of protocols among the included studies, from single infusions to multi-round perfusions. This variability suggests that the therapeutic effect may be more than a one-time event. For example, some studies noted that positive outcomes, such as improved menstrual flow and endometrial regeneration, were transient or began to wane months after the initial treatment. This supports the hypothesis that the beneficial effects are primarily driven by short-lived paracrine signals from the stem cells, rather than long-term engraftment. To achieve lasting results, future research should focus on optimizing treatment protocols. A single dose might initiate a regenerative response, but repeated administrations could be necessary to provide a sustained supply of paracrine factors, leading to more durable endometrial repair. Conversely, excessively frequent administration could be ineffective or lead to complications. The ideal frequency likely depends on the specific cell type and the severity of the uterine damage. Therefore, future studies should move toward standardized, multi-dose protocols to establish evidence-based guidelines for administration frequency, ultimately leading to more robust and lasting therapeutic benefits for patients with intrauterine adhesions.

## Limitations

Despite the valuable insights generated by this meta-analysis, several limitations warrant consideration when interpreting the findings. Firstly, the overall quality of evidence is impacted by the methodological shortcomings of the included studies. A substantial proportion of studies were non-randomized, open-label, and exhibited a high risk of bias across multiple domains, including random sequence generation, allocation concealment, and blinding of participants, personnel, and outcome assessors. This inherent methodological variability and lack of rigorous blinding may introduce performance, detection, and selection biases, potentially overestimating treatment effects. Secondly, the relatively small number of studies and observations for several key outcomes, particularly implantation rate, cumulative biochemical pregnancy, and ectopic pregnancy, restricts the statistical power to detect subtle differences or true underlying heterogeneity. Subgroup analyses, while informative, are limited by the small sample sizes within each category, making it challenging to draw definitive conclusions. The most significant limitation is the substantial heterogeneity in key outcomes like live birth and clinical pregnancy rates. This variability complicates the interpretation of our pooled results and likely stems from differences in study design, patient populations, intervention methods, and outcome measures. For example, patient age, disease severity, and the specific type of stem cell and delivery method used varied greatly across studies. While our subgroup analyses by disease type and intervention method attempted to explore these differences, the limited number of studies within each subgroup prevented us from drawing definitive conclusions. This highlights the urgent need for larger, well-designed, and standardized clinical trials to confirm these promising findings and establish clear treatment protocols. Thirdly, despite subgroup analyses by disease type and intervention method, residual heterogeneity remained significant for cumulative live birth (I^2^ = 46.2%) and cumulative clinical pregnancy (I^2^ = 36.9%). This suggests that unmeasured factors, such as variations in specific cell preparation protocols, dosages, routes of administration, concomitant treatments, patient age, or baseline endometrial characteristics, may contribute to outcome variability. Finally, the meta-analysis primarily included studies from China, with only a few from other countries, potentially limiting the generalizability of the findings to broader populations. The lack of long-term follow-up data for all outcomes further limits our understanding of the sustained efficacy and long-term safety of these interventions.

## Conclusion

This meta-analysis provides compelling evidence that stem cell therapy holds significant promise for improving fertility and pregnancy outcomes in patients with IUA and Asherman's Syndrome, particularly when compared to conventional treatments. The findings underscore that the success of stem cell interventions is significantly influenced by both the underlying disease type and severity and the specific stem cell intervention method. Autologous stem cells delivered with a scaffold/matrix appear to offer the most promising outcomes for cumulative live birth and clinical pregnancy, likely by enhancing cell retention, viability, and creating an optimal regenerative microenvironment. Conversely, the consistent lack of heterogeneity observed for early spontaneous abortion and ectopic pregnancy rates across diverse stem cell interventions suggests that these specific adverse events may be less directly influenced by the variations in stem cell therapy itself. Instead, these outcomes are likely predominantly driven by other factors such as embryonic chromosomal abnormalities (for spontaneous abortion) or tubal pathology (for ectopic pregnancy), which lie largely outside the primary mechanistic scope of uterine-targeted stem cell interventions.

## Data Availability

All data generated or analyzed during this study are included in this manuscript. Further inquiries should be directed to the corresponding author.

## Abbreviation

IUA: Intrauterine adhesions
CI: Confidence interval
OR: Odds ratio
MSCs: Mesenchymal stem cells
MeSH: Medical Subject Headings
RCTs: Randomized controlled trials
